# Platinum-based therapeutics as emerging multi-modal radiosensitizers in glioblastoma treatment

**DOI:** 10.1016/j.addr.2026.115811

**Published:** 2026-02-13

**Authors:** Ananya H. Elati, Erika W. Davies, Mark V. Mishra, Jeffrey A. Winkles, Graeme F. Woodworth, Anthony J. Kim

**Affiliations:** a Department of Neurosurgery, University of Maryland School of Medicine, Baltimore, MD 21202, United States; b University of Maryland Marlene and Stewart Greenebaum Comprehensive Cancer Center, Baltimore, MD 21201, United States; c Fischell Department of Bioengineering, A. James Clark School of Engineering, University of Maryland, College Park, MD 20742, United States.; d Department of Radiation Oncology, University of Maryland School of Medicine, Baltimore, MD 21201, United States.; e Center for Vascular and Inflammatory Diseases, University of Maryland School of Medicine, Baltimore, MD 21201, United States.; f Department of Diagnostic Radiology and Nuclear Medicine, University of Maryland School of Medicine, Baltimore, MD 21201, United States; g Department of Pharmacology, University of Maryland School of Medicine, Baltimore, MD 21201, United States.

**Keywords:** Glioblastoma (GBM), Platinum, Radiosensitization, Nanoformulations, Focused ultrasound, Drug delivery, Multimodal therapy

## Abstract

Glioblastoma (GBM) is a highly lethal brain cancer with poor patient prognosis and limited treatment options. Standard-of-care therapy involves maximally safe surgical resection followed by radiation therapy (RT) in combination with the alkylating agent temozolomide (TMZ) to treat the residual and brain-invading components of the disease. While RT remains a central part of definitive treatment, its efficacy is limited by intrinsic and acquired radiation resistance, making long-term tumor control and survival rare. While TMZ provides modest anti-tumor benefits, tumor resistance to TMZ and lack of synergy with RT underscore the critical need to identify novel therapeutic strategies that can enhance RT efficacy. Platinum-based chemotherapeutics have emerged as promising radiosensitizers due to their ability to augment RT-induced DNA double-strand breaks, while also triggering oxidative stress, endoplasmic reticulum stress, immunogenic cell death, and apoptosis. Such multi-modal mechanisms of action may propel platinum-based drugs to the forefront of GBM treatment and overcome the adaptive survival pathways that underlie radiation resistance. This is particularly the case as advances in drug delivery strategies, such as liposome- and nanocomplex-based formulations, are being developed to improve brain penetration, tumor accumulation, and reduce systemic toxicity-critical challenges that have historically limited the clinical application of platinum-based drugs in GBM. This review provides clinical relevance, mechanisms, delivery strategies, challenges, and recent advancements in the use of platinum compounds as radiosensitizers for GBM treatment, highlighting key preclinical studies, clinical observations, and future directions.

## GRAPHICAL ABSTRACT

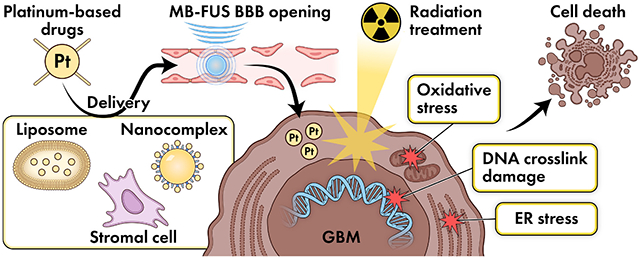

## 1. Introduction

Glioblastoma (GBM) is the most aggressive and common primary brain tumor in adults and is distinguished by its rapid growth, marked brain infiltration, and acquired resistance to treatments [1]. GBM comprises ~47% of all primary malignant brain tumors, with a worldwide incidence rate of 3.22 per 100,000 individuals [2]. The current standard-of-care treatment includes maximally safe surgical resection to remove the tumor and improve neurological function, followed by concurrent radiation therapy (RT) and oral temozolomide (TMZ) chemotherapy to treat the residual brain-invading tumor cells inevitably left behind after surgery [3]. However, this treatment approach has limited efficacy, with a median survival of less than 18 months from patient diagnosis [4].

This poor prognosis can be attributed to the highly invasive and treatment-resistant nature of GBM, which makes complete resection unfeasible and leads to frequent, rapid tumor recurrence [5]. RT remains a central part of GBM treatment, but radiation resistance makes long-term tumor control and survival rare [6]. Moreover, the efficacy of TMZ is often limited by intrinsic or acquired resistance that develops in over 50% of GBM patients [7]. One important resistance mechanism is driven by the epigenetic regulation of the DNA repair enzyme, O6-methylguanine-DNA methyltransferase (MGMT), where promoter methylation status serves as a prognostic indicator and predictive biomarker of TMZ responses [7]. Specifically, patients with an unmethylated MGMT promoter (~70% of cases) express higher levels of the MGMT repair enzyme, which removes TMZ-induced DNA adducts, thereby reducing this drug’s therapeutic efficacy.

Thus, there is a critical need to identify novel therapeutic strategies that can enhance multi-modal RT efficacy for GBM patients. One class of therapeutics that is amassing growing interest for the treatment of GBM is platinum-based drugs due to their highly cytotoxic, multi-modal mechanisms of action and ability to covalently bind DNA, avoiding the epigenetic regulation of MGMT and development of resistance. Such properties can potentially combine to overcome the intrinsic radioresistance of GBM cells and improve chemo-radiation efficacy.

## 2. Current status of radiation therapy in GBM

RT is a major cornerstone in the treatment of GBM. The standard-of-care RT regimen for GBM typically delivers 60 Gy in 30 fractions over six weeks (5 days per week), a protocol associated with improved survival outcomes in patients under 65 years of age, compared to alternative regimens [8]. In older patients or patients with a poor performance status, shorter and hypofractionated regimens, such as 40 Gy delivered in 15 fractions over three weeks or 50 Gy delivered at 1.8 Gy per fraction, are often preferred, offering comparable survival with reduced toxicity [9,10]. Nevertheless, median survival following all treatment paradigms remains under two years. Despite the widespread use of RT to treat GBM, its efficacy is significantly impeded by the inherent radioresistance of a subset of GBM cells, resulting in poor prognosis and high recurrence rates [6].

GBM cells exhibit various mechanisms that contribute to radioresistance such as tumor heterogeneity, increased activation of DNA damage repair pathways, hypoxia-induced changes, and metabolic reprogramming [6]. These factors collectively enable the survival and proliferation of GBM cells even after exposure to radiation. To overcome this challenge, recent studies have explored advances in RT techniques and novel sensitization strategies (*e.g*. hyperthermia [11]). Stereotactic radiosurgery and dose escalation beyond 60 Gy have aimed to improve GBM tumor targeting and progression-free survival but have not significantly extended overall survival [12,13]. Recent studies have also explored the incorporation of targeted pharmacological interventions with RT. For example, the DNA alkylator seco-duocarmycin SA (sDSA) demonstrated enhanced cytotoxicity when combined with proton radiation, compared to either intervention alone [14]. Targeted therapies that exploit radiation-induced metabolic shifts are also under investigation as potential radiosensitizing strategies [15].

Combining RT with immune checkpoint inhibitors has shown promise in preclinical studies, suggesting there may be synergistic effects through modulation of the tumor microenvironment [16]. However, a recent phase II clinical trial investigating a dual immune checkpoint blockade in MGMT-unmethylated GBM patients found no improvement in progression-free and overall survival compared to the standard-of-care RT + TMZ regimen [17]. Therefore, to meaningfully overcome GBM’s radioresistance and improve clinical outcomes, the incorporation of effective radiosensitizers will likely be essential.

## 3. Radiosensitizers for GBM treatment

Radiosensitizers are emerging as a promising and viable strategy to enhance RT efficacy in GBM by selectively increasing tumor cell susceptibility to radiation-induced damage, which has the potential to improve therapeutic outcomes while minimizing damage to surrounding healthy tissues. The development of novel radiosensitization strategies is an important step in overcoming the intrinsic radioresistance of GBM. Targeting specific molecular pathways involved in DNA repair and cellular responses to radiation can significantly amplify radiosensitivity. One approach involves targeting DNA damage repair mechanisms. For instance, the inhibition of DNA-dependent protein kinase (DNA-PK), a critical enzyme in the non-homologous end joining (NHEJ) repair process, has shown significant radiosensitizing effects. In a recent study, the DNA-PK inhibitor peposertib significantly enhanced radiosensitivity in GBM cells by impairing DNA repair and enhancing post-radiation cytotoxicity [18].

Clinically, effective radiosensitization may also allow for the use of lower radiation doses, thereby mitigating RT-associated toxicities. Table 1 provides a comprehensive overview of past and ongoing clinical trials investigating various radiosensitizers for GBM treatment. While many of the candidates demonstrated encouraging results in early-phase trials (such as improved tolerability), most failed to translate into significant survival benefits in the later phases of the trials. For instance, adavosertib exhibited unacceptable toxicity in combination with TMZ and radiation, while others such as erlotinib and dasatinib did not improve overall survival despite promising preclinical data. Notably, the mood stabilizing and anti-epileptic drug valproic acid showed a promising median overall survival of 29.6 months with 86% of the participants demonstrating overall survival at 12 months; however, confirmation of its therapeutic benefit will require validation in a well-powered Phase III clinical trial in GBM patients.

These findings emphasize the need for continued research into the molecular underpinnings of radioresistance and the evaluation of more effective radiosensitizers. Future studies should aim to integrate these radiosensitizers into standard treatment protocols and assess their efficacy in combination with TMZ and radiation, especially in patient subpopulations stratified by biomarkers such as MGMT promoter methylation. This may pave the way for improved therapeutic outcomes in this highly treatment-resistant brain malignancy.

## 4. Platinum-based Drugs in GBM

Among emerging radiosensitizer candidates for GBM, platinum-based compounds have shown considerable potential. There are three FDA-approved platinum-based drugs – cisplatin, carboplatin, and oxaliplatin – that have been cornerstones in the treatment of various malignancies such as ovarian, breast, and colorectal cancers for decades [19]. These drugs function by forming DNA adducts wherein the platinum atoms bind to and form crosslinks with DNA that inhibit DNA replication and transcription [20]. The subsequent DNA damage results in the upregulation of transcription-coupled repair mechanisms and if the lesion cannot be repaired, it induces a cascade of events leading to cancer cell apoptosis [20,21].

Platinum-based chemotherapy has been shown to improve overall survival and progression-free survival outcomes when combined with RT in various solid tumors including head and neck cancer and nasopharyngeal cancer as a standard-of-care treatment [22,23]. These benefits are attributed to the ability of platinum drugs to disrupt DNA repair processes and amplify radiation-induced cellular damage [24,25]. Through these mechanisms, platinum-based therapeutics may enhance radiation efficacy, reduce tumor recurrence, and potentially prolong overall survival in GBM patients.

Despite significant efficacy in treating non-central nervous system (CNS) cancers, translation to GBM has faced challenges, most notably severe systemic toxicities, and dose-limiting neurotoxicity [20]. Recent advancements have led to the development of novel platinum complexes and delivery systems to enhance their therapeutic efficacy while also minimizing adverse effects [24]. For instance, platinum (IV)-based prodrugs and nanoformulations are being explored to improve tumor specificity and reduce off-target toxicity [24]. These innovations aim to overcome the limitations of traditional platinum drugs and harness their full potential in cancer therapy. However, a deeper mechanistic understanding of how platinum compounds contribute to radiosensitization could inform the rational design of more effective combination therapies. Such insights are important to overcoming GBM’s inherent radioresistance and translating platinum-based radiosensitizers to the clinic.

## 5. Mechanisms of platinum-based radiosensitization

### 5.1. DNA damage enhancement

**Fig. 1.**
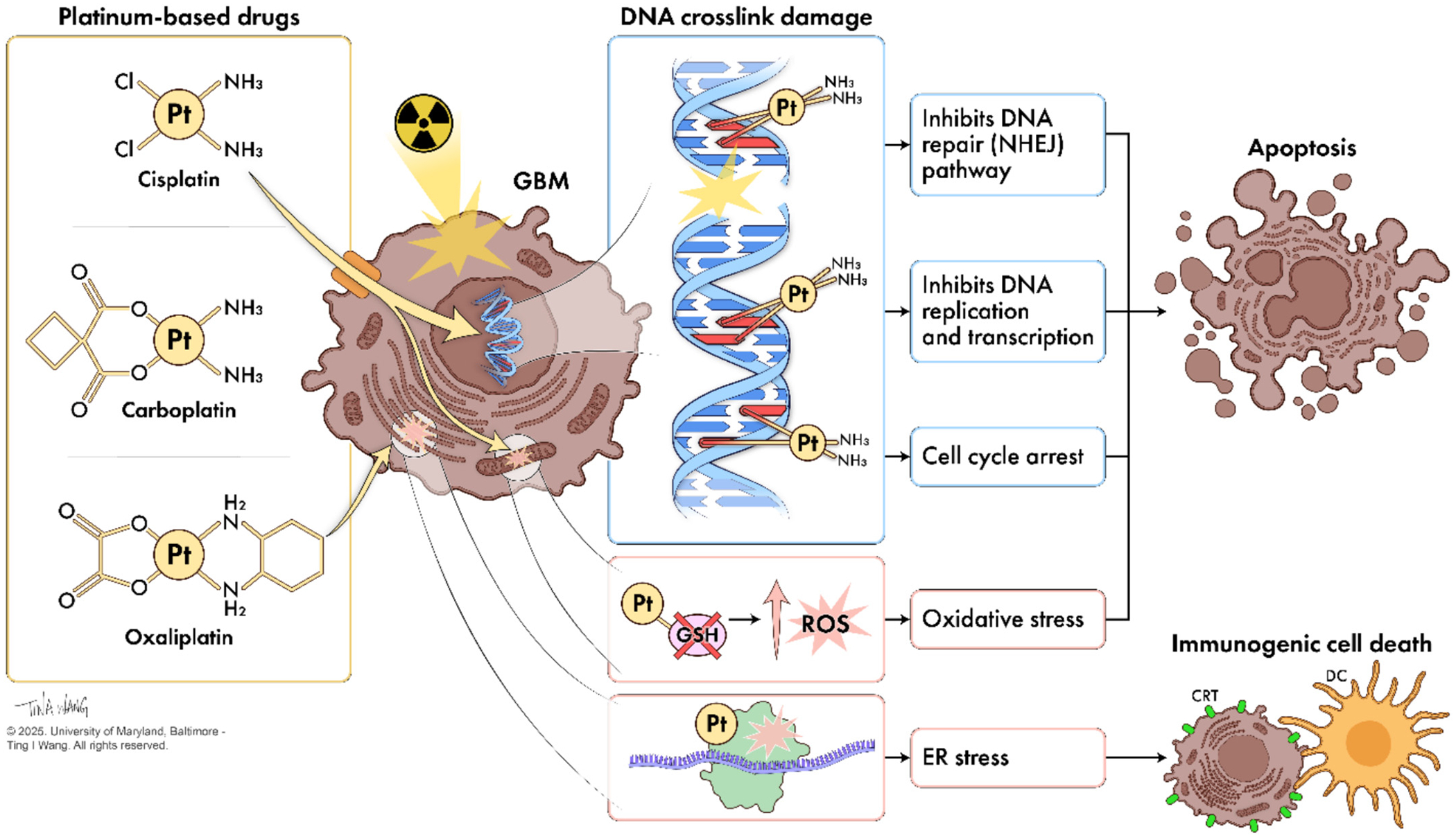
Mechanisms of platinum compound-based radiosensitization. The three FDA-approved platinum drugs can radiosensitize GBM cells through various mechanisms such as DNA damage enhancement, inhibition of DNA repair pathways, cell cycle arrest, and increases in oxidative stress. These mechanisms contribute to apoptosis and have been described in further detail in this section.

One of the primary mechanisms through which platinum-based agents exert their radiosensitizing effects involves the induction of DNA damage which occurs through covalent binding between platinum and purine bases, forming DNA cross-links that interfere with DNA replication and transcription (Fig. 1), leading to the accumulation of DNA double strand breaks (DSBs). When used in combination with ionizing radiation, platinum agents significantly increase the formation of lethal DSBs, exacerbating radiation-induced DNA damage. This overwhelms the cell’s repair mechanisms and contributes to enhanced cytotoxic effects [24]. The presence of high atomic number (Z) elements like platinum in these drugs also contributes to radiosensitization by increasing the emission of ionizing photoelectrons and Auger electrons, which further augments DNA damage [26].

### 5.2. Cell cycle and stress response modulation

DNA damage activates the repair machinery of the cell and causes cell cycle arrest at specific control checkpoints to repair DNA damage and prevent cell cycle progression [25]. GBM cells are inherently radioresistant due to their ability to effectively repair radiation-induced DNA damage, largely through upregulation of genes involved in the DNA damage response. This enables their survival and proliferation, leading to tumor cell infiltration and GBM recurrence. Platinum drugs can potentially address this challenge due to their ability to arrest cells in the radiosensitive phases of the cell cycle (especially the G2/M phase), enhancing their susceptibility to radiation damage [27]. This reduces the probability of DNA damage repair, inducing cancer cell apoptosis and enhancing the efficacy of radiation treatment.

Another mechanism by which platinum drugs enhance radiosensitivity is through the modulation of cellular stress responses. Platinum drugs can induce oxidative stress within GBM cells, which can then potentiate the DNA damage caused by radiation. Platinum-based drugs generate reactive oxygen species (ROS) by directly catalyzing redox reactions, forming DNA adducts in mitochondrial DNA that impair replication and transcription and damage mitochondrial proteins, and through activation of specific signaling pathways that promote ROS generation. For example, a platinum-maurocalcine conjugate has been shown to interact with the ROS-ERK/AKT-p53 pathway, leading to enhanced apoptosis in GBM cells [28]. This compound induces intracellular oxidative stress, which in turn activates p53, a key regulator of the cell cycle and apoptosis. The inhibition of survival pathways such as AKT and ERK (which are aberrantly activated in various human cancers) further sensitizes the cells to radiation-induced damage [28]. This study highlights the potential of targeting specific signaling pathways to improve the efficacy of platinum-based radiosensitization.

Platinum drugs further disrupt cellular redox balance by decreasing the availability and/or function of antioxidants, such as glutathione (GSH). Platinum binds GSH to form platinum-GSH conjugates which then deplete GSH and increase ROS accumulation [29]. This can lead to the generation of single strand breaks (SSBs), abasic sites, sugar moiety modifications, and deaminated adducted bases that can induce mitotic failure and result in the activation of apoptotic pathways, which is crucial for the induction of cell death in response to DNA damage [28,30]. The combination of platinum drugs with radiation can thus lead to a more pronounced activation of various mitochondrial and death receptor pathways, resulting in increased apoptosis of GBM cells.

### 5.3. Inhibition of DNA repair pathways

Another mechanism by which platinum compounds can enhance radiation efficacy is through the inhibition of DNA repair pathways. One study explored the synergistic effects of combining cisplatin with RT in ovarian cancer treatment [31]. It investigated the mechanism behind this synergy, focusing on the role of the NHEJ pathway, which is important for catalyzing the repair of DNA DSBs. The results from this study demonstrated that cisplatin enhances the sensitivity of cancer cells to RT by inhibiting the NHEJ pathway [31]. This inhibition is a result of the cisplatin-DNA lesions near the DSB terminus which prevent the NHEJ repair process from occurring. Notably, this synergistic interaction is evident in cells proficient in NHEJ but not in NHEJ-deficient cells, indicating that the presence of a functional NHEJ pathway may be necessary for cisplatin sensitization to RT [31]. These findings suggest that targeting the NHEJ pathway could be a potential strategy to enhance the effectiveness of cisplatin and RT in GBM therapy.

Another study found that platinum complexes can inhibit key DNA repair and replication proteins like Ku70 and topoisomerase IIα, leading to increased DNA damage [32]. This is marked by elevated phosphorylation of histone H2AX on serine 139 (γ-H2AX), checkpoint kinase 1/2 (Chk1/2) phosphorylation, p53 activation, and cell cycle arrest [32]. The downregulation of Ku70 and the inhibited formation of Ku70 foci suppresses DNA repair and provokes a NHEJ response, leading to apoptosis in a variety of cancer cell lines [32]. By impairing the cell’s ability to repair DNA lesions, platinum drugs can significantly amplify the cytotoxic effects of radiation.

### 5.4. Modulation of the tumor microenvironment

Tumor hypoxia is a key driver of radioresistance, and multiple studies have demonstrated the ability of platinum drug-based nanoplatforms to counteract tumor hypoxia. They do so by catalyzing the decomposition of tumor endogenous hydrogen peroxide into oxygen, thereby increasing tumor oxygenation [33–36]. Porous platinum nanoparticles, platinum-iron nanoparticles, and platinum-functionalized nanocomposites have all been shown to sufficiently elevate oxygen levels enough to amplify RT-induced DNA damage and ROS production [33–36]. Studies have also shown that the inhibition of hypoxia-driven survival pathways such as hypoxia-inducible factor-1α (HIF-1α) signaling can suppress effects linked to radioresistance and immunosuppression [36]. Amelioration of tumor hypoxia not only enhances the cytotoxic effects of RT but also inhibits HIF-1α-mediated angiogenesis and metabolic rewiring, promoting cell cycle arrest and restoring radiosensitivity [37].

Platinum agents can also help reshape the tumor microenvironment (TME) through modulation of tumor-associated macrophages (TAMs). Hypoxia skews macrophages toward an immunosuppressive M2-like macrophage phenotype, promoting tumor progression [38]. Through alleviation of hypoxia, platinum nanoplatforms can help shift TAMs from the pro-tumoral M2-like phenotype to the anti-tumoral and pro-inflammatory M1-like phenotype, enhancing anti-tumor immunity [38]. Several studies (including biogenic platinum nanocomplexes and biotin receptor-targeting oxaliplatin-loaded lipid nanoparticles) also show enhanced cytokine production and improved dendritic cell maturation, reversing immune suppression [33,39]. Such immunologic changes synergize with RT by increasing cytotoxic T cell infiltration and reducing regulatory T cell populations [33,39]. These findings suggest that platinum-based therapeutics function not only as cytotoxic agents and radiosensitizers, but also as potent immunomodulators, where TME remodeling effects strengthen the biological rationale for exploring platinum agents within combination immuno-radiotherapy paradigms.

### 5.5. Induction of endoplasmic reticulum stress and immunogenic cell death

Interestingly, oxaliplatin distinguishes itself from cisplatin and carboplatin by additional mechanisms of action in GBM, particularly through its ability to induce endoplasmic reticulum (ER) stress, a cellular stress response caused by an accumulation of unfolded proteins in the ER, and immunogenic cell death (ICD) [20,40]. While all three platinum agents are capable of triggering ER stress, oxaliplatin’s cytotoxicity is not primarily mediated through DNA damage, unlike cisplatin and carboplatin, which function by forming DNA adducts that disrupt replication and transcription. Uniquely, oxaliplatin exerts its effects by inducing ribosome biogenesis stress, a distinct cellular stress pathway that affects protein synthesis and ribosomal function, intersecting with ER stress response, which then leads to immunogenic and apoptotic cell death [41,42]. This unique action of oxaliplatin may influence how it sensitizes GBM cells to radiation, potentially offering a different and complementary mechanism to the DNA-damage-induced ER stress seen with cisplatin and carboplatin [42]. This mechanistic diversity could inform the selection of platinum drugs for radiosensitization strategies in GBM, depending on the desired cellular stress pathway to exploit. These multimodal mechanisms may also potentially offer therapeutic advantages in tumors with high DNA repair capacity or intrinsic resistance to traditional DNA damaging therapies.

The induction of ICD by oxaliplatin is a specific type of regulated cell death that activates an adaptive immune response against tumor cells [20,43]. A hallmark of ICD is the translocation of calreticulin (CRT) to the cell surface which signals engulfment by dendritic cells and is dependent on ER stress through activation of the protein kinase R-like ER kinase (PERK) pathway [43]. Unlike oxaliplatin, cisplatin fails to induce CRT exposure despite increasing the extracellular release of ATP and HMGB-1 (two ICD markers), indicating its limited capacity to elicit a full immunogenic response [20,43]. Thus, oxaliplatin not only enhances radiosensitivity through ER stress and ribosomal disruption but may also prime tumors for immune-mediated clearance, a feature particularly valuable in the immunosuppressive microenvironment of GBM.

These findings highlight oxaliplatin’s multi-modal mechanisms of action which involve ER stress, ribosomal disruption, and immunogenicity to offer a distinct and potentially more effective radiosensitization and anti-tumor profile compared to other platinum agents. These mechanistic insights support further evaluation of oxaliplatin in GBM combination therapies, especially in strategies integrating radiation with immunotherapy or ER stress modulators. While platinum drugs demonstrate strong radiosensitization properties through a variety of mechanisms, their high toxicity and inability to cross the BBB limits their use in GBM treatment. To address these challenges, ongoing preclinical efforts have focused on the design of unique drug formulations and innovative delivery strategies to enable the use of platinum compounds as radiosensitizers for GBM treatment.

## 6. Advanced delivery strategies for platinum-based drugs in GBM

### 6.1. Cell-based delivery systems

A recently published study investigated the use of mesenchymal stromal cells (MSCs) as a delivery system for a novel platinum-based drug, Pt-8AQ, in GBM treatment [44]. Key findings from this study show that Pt-8AQ, more stable than cisplatin, is successfully incorporated into MSCs and released into the extracellular milieu, with nearly 50% released 48 h post-intratumoral administration [44]. Delivery of Pt-8AQ through MSCs exhibits greater anticancer activity against U87 GBM tumors compared to the free drug, with lower IC50 values [44]. This improved efficacy is due to the ability of MSCs to preferentially migrate toward sites of injury and inflammation (such as GBM tumors), making them attractive candidates for systemic targeted drug delivery, with the capacity to actively target tumor tissue and release the drug where it is most needed, maximizing its therapeutic effect [45].

The use of MSCs as a delivery vehicle also improves the safety profile of platinum agents by maximizing intratumoral drug concentrations, limiting exposure to healthy tissues, and lowering the risk of adverse systemic toxicity effects associated with them such as neurotoxicity and nephrotoxicity [46]. MSCs also appear to possess relative resistance to the cytotoxic effects of platinum drugs, allowing them to function effectively without significant loss of viability [47]. Although this study did not incorporate radiation, its findings suggest a promising delivery system whose safety and biodistribution could be investigated in future studies and potentially adapted for the systemic delivery of platinum-based radiosensitizers.

Additional evidence supporting the feasibility of MSC-mediated delivery in GBM comes from a study demonstrating robust MSC migration in multiple GBM orthotopic models [48]. Human MSCs (hMSCs) injected intracerebrally or systemically were shown to extravasate, localize within GBM tumors, reduce tumor proliferation and vascularization, and significantly extend survival, emphasizing their strong potential as delivery platforms for platinum drugs [48]. Although the paclitaxel-loaded hMSCs did not show significantly enhanced antitumor efficacy relative to the unloaded hMSCs, these findings suggest that cargo selection is critical. Given their multimodal radiosensitizing properties and MGMT-independent mechanisms, platinum agents may represent more effective payloads for MSC-mediated delivery to GBM tumors.

Due to the tumor-homing capacity of neutrophils and their inherent ability to cross physiological barriers like the BBB, they represent another cell type of interest as delivery platforms for GBM [49]. A preclinical study showed that a chimeric antigen receptor (CAR)-neutrophil platform delivering TME-responsive nanodrugs to GBM was able to traverse the BBB and exhibited potent antitumor efficacy, drug delivery specificity, and prolonged survival in orthotopic models [49]. These results, while not specific to platinum drugs, highlight the potential of neutrophils as a promising and versatile cell-based delivery strategy for GBM radioimmunotherapy treatments utilizing platinum drugs, related to superior anti-GBM activity and diminished off-target effects. Future studies will be critical to determine whether this approach can fully exploit the radiosensitizing potential of platinum agents in GBM.

### 6.2. Liposomal-based delivery formulations

Liposomal delivery systems have emerged as a promising approach to enhance the therapeutic efficacy and safety profile of platinum-based drugs, particularly when combined with RT. *In vitro* studies conducted with F98 glioma cells have demonstrated that liposomal platinum compounds significantly amplify the cytotoxic effects of ionizing radiation [50]. For instance, a liposomal cisplatin formulation (Lipoplatin^™^) enhances cellular uptake by three-fold and increases radiosensitization by 14-fold relative to free cisplatin [50]. Conversely, a liposomal oxaliplatin formulation (Lipoxal^™^) exhibits synergistic effects with radiation only at concentrations exceeding its IC50, which may be due to its lower cell uptake compared to Lipoplatin^™^, highlighting the value in developing targeted formulations [50].

*In vivo* studies investigating free platinum drugs in combination with RT have also demonstrated promising results. Carboplatin administered *via* convection-enhanced delivery (CED) followed by a 15 Gy radiation dose significantly prolonged the median survival times of F98 glioma-bearing Fischer rats from 38.5 days to 54 days [51]. Mechanistically, these formulations improve drug delivery and retention at the tumor site, maximizing the therapeutic impact of radiation. Importantly, the timing of radiation relative to drug delivery influenced outcomes; a 24-h interval between CED and radiation yielded better survival than a 4-h interval, despite higher platinum-DNA adduct levels observed at the earlier time point [51].

Building upon these results, liposomal formulations of carboplatin were investigated to evaluate toxicity effects and tumor uptake, with the aim of not only enhancing radiosensitization but also reducing systemic toxicity and improving brain tissue penetration. Cationic liposomal formulations of carboplatin exhibit increased cytotoxicity *in vitro* compared to free carboplatin while *in vivo* studies showed that negatively charged PEGylated liposomes achieved more favorable therapeutic outcomes [52]. These formulations appeared to diffuse more effectively within the TME, overcoming the limitations of the BBB and contributing to extended survival times in animal models. CED of Lipoxal^™^ was found to reduce toxicity while still maintaining a similar median survival time in F98 glioma-bearing Fischer rats relative to free oxaliplatin [53]. The MTD of Lipoxal^™^ also increased three-fold, confirming the ability of liposomal formulations to significantly increase drug dosing and potentially, efficacy [53].

Overall, liposomal platinum formulations demonstrate significant potential in improving GBM treatment paradigms when combined with RT. These advances emphasize the importance of optimizing liposomal properties, such as charge and PEGylation, as well as the timing of drug delivery to maximize therapeutic efficacy. Ongoing research aims to refine these formulations and validate their clinical safety and efficacy profiles, paving the way for more effective GBM treatment strategies. Further investigations and clinical trials will be critical to translating these promising preclinical findings into standardized treatment regimens. Several of these liposomal platinum drugs such as LiPlaCis and MBP-426 have already undergone clinical testing in non-CNS cancers, as outlined in Table 2, which summarizes a list of clinical trials evaluating platinum drug-based nanoformulations. Although these trials are not GBM-specific, their design and outcomes provide valuable insights that could inform future clinical applications in GBM tumors.

Clinical evaluation of these platinum-based nanoformulations demonstrates that delivery platform design strongly influences pharmacokinetics, toxicity, and therapeutic responses which are key factors for determining their suitability as GBM radiosensitizers. Liposomal cisplatin formulations such as LiPlaCis and sustained release lipid inhalation targeting (SLIT) cisplatin emphasize improved tolerability compared to conventional cisplatin, with clinical studies reporting manageable toxicity profiles and measurable clinical benefit in subsets of patients. For example, LiPlaCis achieved a median overall survival of 50 weeks in metastatic breast cancer patients, while SLIT cisplatin demonstrated minimal intravenous cisplatin-associated toxicities and produced sustained therapeutic benefit in 3 of 8 osteosarcoma patients [54]. These findings suggest that liposomes can effectively mitigate systemic toxicity and maintain pharmacologic exposure - a property desirable for radiosensitization where cumulative toxicity can limit combined-modality regimens. However, clinical responses remain varied, likely reflecting limited active targeting and the dependence of liposomal accumulation on tumor vascular permeability.

Next-generation delivery systems, including stealth (PEGylated) liposomes (SPI-77) and micellar nanocomplexes (NC-6004), highlight the performance trade-offs associated with deeper pharmacokinetic optimization. SPI-77 substantially reduced cisplatin-associated toxicities but failed to demonstrate adequate antitumor efficacy in a Phase II recurrent ovarian cancer study, emphasizing the idea that prolonged circulation alone does not guarantee sufficient intratumoral drug release or clinical benefit [55]. This limitation is particularly instructive for GBM, where nanocarriers must not only traverse the BBB but also achieve timely intracellular platinum release within the narrow therapeutic windows imposed by fractionated radiotherapy. Therefore, overly high carrier stability without controlled drug release property risks drug sequestration without functional radiosensitization.

In contrast, NC-6004’s micellar platform achieved improved tolerability with encouraging disease control rates across solid tumor studies (tumor shrinkage and stable disease in 55–70% of patients), though one head and neck cancer trial was terminated, likely reflecting challenges in dose optimization and tumor-specific uptake. In the context of GBM, such obstacles may be mitigated through local delivery strategies or systemic administration combined with focused ultrasound-mediated BBB opening, enabling enhanced tumor uptake while avoiding the impracticality of frequent intracranial injections. Importantly, MBP-426 illustrates the added value of ligand-mediated targeting: transferrin receptor-directed liposomes demonstrated favorable tolerability and efficient delivery in Phase I studies, establishing receptor-targeted nanocarriers as promising platforms for tumors with defined overexpression profiles. Given the overexpression of multiple receptor targets in GBM, including transferrin and Fn14, ligand-targeted platinum nanocarriers represent a particularly promising strategy to improve tumor selectivity, enhance radiosensitization, and reduce off-target platinum accumulation.

Collectively, these clinical outcomes suggest that optimal strategies for future platinum-based radiosensitizers will require platforms that balance enhanced tumor penetration, controlled drug release, and minimized systemic toxicity. Conventional liposomes provide safety advantages, but targeted liposomes and polymeric micelles offer greater control over pharmacokinetics and tumor selectivity. For GBM specifically, where the BBB severely restricts delivery, formulations incorporating active targeting, sustained-release mechanisms, or CED compatibility are likely to provide the greatest therapeutic leverage. These comparative findings highlight the need to integrate delivery design with GBM’s biological constraints to maximize the safety and efficacy profiles of platinum radiosensitizers.

### 6.3. Polymeric nanocomplex-based delivery formulations

Nanocomplex platforms offer an advanced approach for targeted platinum drug delivery, enhancing their radiosensitizing properties while mitigating systemic toxicity [56]. One innovative strategy involves the use of cisplatin-tethered gold nanospheres. A preclinical study performed on patient-derived treatment-resistant GBM cells showed that cisplatin-tethered gold nanospheres were able to improve the intracellular delivery of cisplatin, leading to increased DNA damage and apoptosis [57]. As with platinum, the high atomic number of gold enhances radiosensitization, contributing to a synergistic increase between cisplatin and RT-mediated cytotoxicity, resulting in the complete ablation of GBM tumor cells *in vitro* [57]. This approach not only improves the efficacy of cisplatin but also reduces its systemic toxicity. While these findings are compelling, further *in vivo* validation is needed to support clinical translatability.

Another study which investigated the encapsulation of carboplatin in biodegradable poly(lactic-*co*-glycolic acid) (PLGA) nanocomplexes demonstrated marked alteration in its therapeutic profile. Results from the study showed that carboplatin-loaded PLGA nanocomplexes substantially enhanced *in vitro* cytotoxicity at 24–48 h compared to equivalent free drug concentrations [58]. This formulation also caused far less neurotoxicity than free carboplatin, preserving both neuronal and glial cell integrity after 72 h of exposure [58]. Promising data was also observed *in vivo*, with CED of PLGA carboplatin resulting in broad distribution throughout the striatum and increased tissue retention at lower infused concentrations relative to free drug [58]. These findings suggest that PLGA encapsulation not only enhances tumor exposure to the cytotoxic effects of platinum drugs but also reduces off-target neuronal damage - a critical consideration for GBM drug delivery systems.

Beyond improving cytotoxicity in tumors and reducing neurotoxicity, PLGA nanocomplex-mediated delivery *via* CED also offers other advantages. The use of CED as an administration method bypasses the BBB, permitting localized, high-concentration delivery with minimal systemic exposure. The biodegradable nature of PLGA ensures that after drug release, polymer breakdown products (lactic and glycolic acid metabolites) are cleared through established interstitial fluid drainage pathways without long-term tissue accumulation, providing an important safety feature when repeatedly dosing in the CNS [58].

### 6.4. Focused ultrasound-mediated blood-brain barrier (BBB) opening

**Fig. 2.**
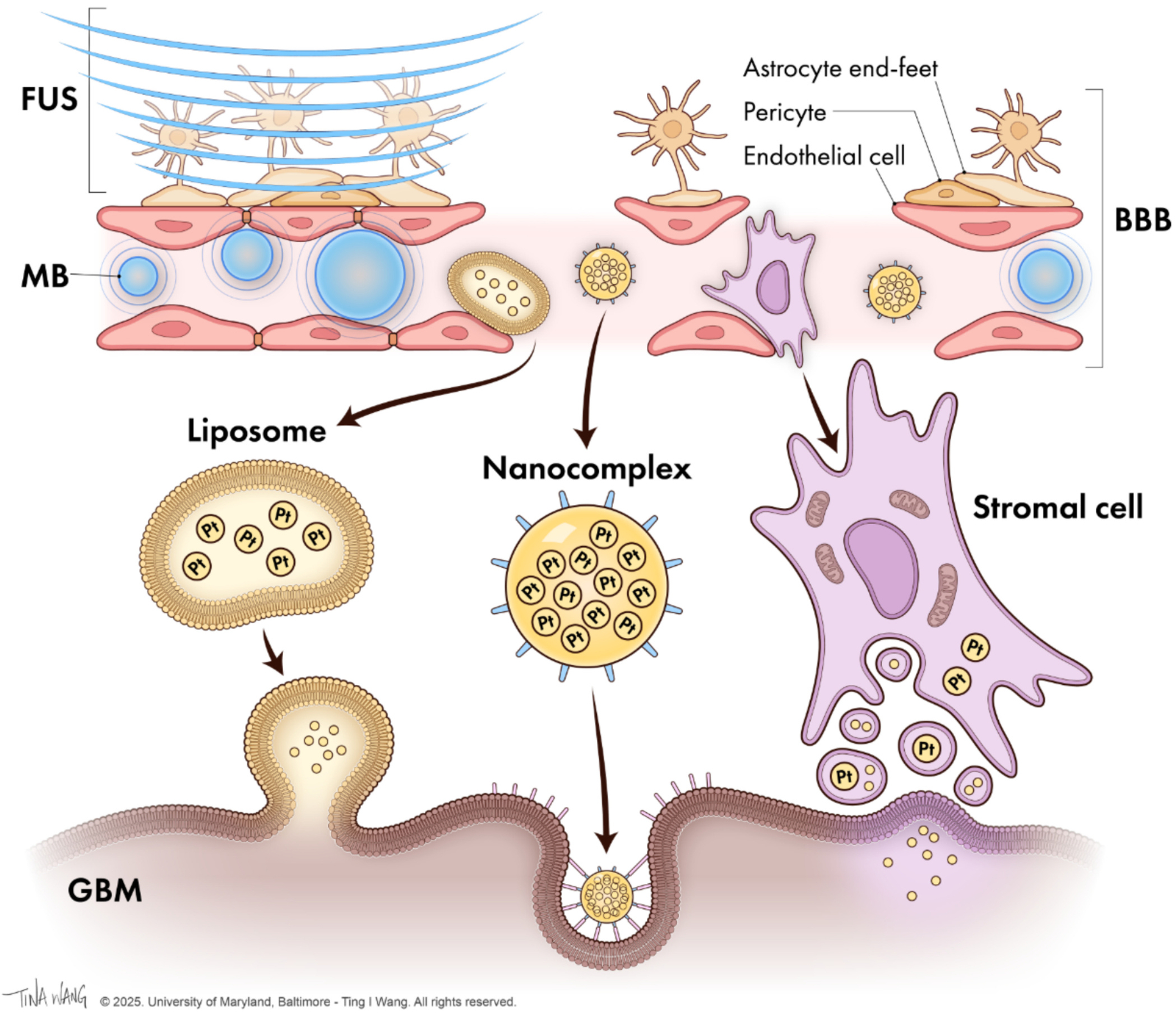
Use of MB-FUS to deliver platinum-based drugs across the BBB. Low-frequency pulsed ultrasound waves are used to excite microbubbles (MBs) and induce oscillations. These oscillations then disrupt the tight junctions of the BBB’s endothelial cells, resulting in the temporary opening of the BBB, which allows platinum-based drugs to be delivered to GBM tumors through liposomes, nanocomplexes, or stromal cells.

The data from the *in vivo* CED study of liposomal carboplatin is compelling, but it does not provide a solution to the challenges faced by the systemic delivery of platinum radiosensitizers [52]. One of the primary obstacles in using platinum-based radiosensitizers for GBM treatment is the BBB, which significantly hinders the delivery of therapeutic agents to the tumor site. The BBB acts as a formidable obstacle, preventing many drugs from reaching effective concentrations within the brain tissue [59]. Additionally, systemic side effects of the highly cytotoxic platinum drugs pose a significant challenge, as they can lead to severe toxicity in patients, limiting the dosage and efficacy of the treatment [59]. As illustrated in Fig. 2, microbubble infusion combined with low-intensity focused ultrasound (MB-FUS) is a non-invasive method of transiently increasing the permeability of the BBB. This promising strategy may enhance the systemic delivery of platinum-based drugs to the tumor site [60–62]. This method allows for the targeted release of drugs within the tumor, potentially reducing off-target side effects. The encapsulation of drugs such as doxorubicin in microbubbles has shown efficacy in preclinical studies, emphasizing the potential of MB-FUS to extend to platinum therapeutics for GBM treatment [63].

In another study, cisplatin-gold nanoconjugates were combined with magnetic resonance-guided focused ultrasound (MRgFUS) [64]. This method enhances platinum concentrations in the cell, greatly inhibiting GBM cell growth relative to free cisplatin and increasing DNA damage through γH2AX phosphorylation [64]. Similar data is observed *in vivo*, with the cisplatin-gold nanoconjugate significantly decreasing GBM tumor burden and MRgFUS increasing BBB permeability and enhancing the delivery of cisplatin to the target tumor site [64]. The combination of this nanoformulation with RT shows promising synergy, improving therapeutic outcomes in GBM treatment.

Clinically, several trials are evaluating the feasibility of MB-FUS combined with carboplatin. In the phase I/II clinical trial (NCT03744026), an implanted multi-transducer MB-FUS device (Carthera Inc., SonoCloud-9, France) was investigated for temporary opening of the BBB in patients with recurrent GBM [65]. This trial involved treatment with intravenous delivery of carboplatin and MB-FUS to enhance carboplatin delivery to the tumor site. There was a 5.9-fold increase in peritumoral brain carboplatin levels after sonication relative to non-sonicated regions of the brain, demonstrating the potential to overcome a major limitation of brain tumor chemotherapy [65]. Results from 12 patients showed improved survival outcomes with a median overall survival of 14 months from surgery, surpassing the survival rate of 9–11 months typically seen in this patient population [65]. Building on the success of this trial, a randomized phase III trial, SONOBIRD (NCT05902169), is currently ongoing and aims to compare the efficacy of carboplatin administered *via* MB-FUS to current standard-of-care treatments [65]. These trials provide strong early evidence that MB-FUS is a safe and promising approach for enhancing platinum drug delivery in GBM, but further clinical validation and promising results from the ‘SonoBird’ trial are required to establish its feasibility and impact on patient outcomes.

Although MB-FUS is increasingly being recognized as a powerful strategy to enhance drug delivery in GBM, its optimal integration into multimodal radiosensitization regimens relies critically on treatment sequencing. In a murine diffuse midline glioma model, FUS was administered concurrently with fractionated RT, one week post-RT, and one month post-RT, without added neurotoxicity and with consistent BBB closure by 72 h post-sonication [66]. These results indicate that RT does not impair FUS efficacy and that repeated BBB openings are feasible within a hypofractionated schedule. In another GBM preclinical and clinical study, FUS was administered within 2 h before RT, improving survival over low-dose RT preclinically, and proving safe clinically in GBM patients [67]. Collectively, these preclinical and early clinical studies demonstrate that FUS-mediated BBB opening can be safely performed before, during, or after RT, suggesting substantial flexibility in scheduling [66,67].

The pharmacokinetics of platinum agents further inform optimal sequencing. While data specifically combining platinum drugs with intracranial FUS remain limited, extracranial tumor models can provide important insights. A preclinical study on orthotopic pancreatic tumors found that systemic administration of FOLFIRINOX followed by MB-FUS significantly increased intratumoral platinum accumulation by 2.5-fold within the first few hours after dosing, with measurable elevations detected as early as 1–2 h post-sonication [68]. These findings suggest that FUS-enhanced uptake of platinum drugs administered systemically peaks within a short window, supporting a regimen in which conventional platinum drugs are administered immediately before or during FUS, with sonication scheduled on the same day as selected RT fractions. This strategy allows the several-hour period of peak intratumoral platinum concentration and BBB permeability to overlap with radiation delivery.

For longer-circulating platinum formulations (such as PEGylated liposomal carriers), a delayed FUS schedule may be more effective, since maximal tumoral accumulation may occur several hours post-injection. Optimal clinical protocols will likely require tailoring FUS timing to the pharmacokinetics of the specific platinum formulation and aligning BBB opening with individual radiation fractions to maximize therapeutic synergy while minimizing toxicity.

## 7. Challenges and future directions

An exciting frontier in GBM treatment lies in combining treatment modalities to achieve multi-modal therapeutic efficacy. Platinum-based radiosensitizers with other therapeutic modalities such as immunotherapies and poly(ADP-ribose) polymerase (PARP) inhibitors offer significant opportunities. PARP inhibitors are known to potentiate radiosensitivity by disrupting DNA repair mechanisms, which is a process that may synergize with the DNA-damaging effects of platinum compounds [69,70]. Combining these treatments could enhance the effects of radiosensitization and offer durable treatment responses, leading to better clinical outcomes. The integration of platinum drugs with immunotherapies, particularly immune checkpoint inhibitors, also presents a promising yet underexplored strategy. To fully leverage oxaliplatin’s unique ability to induce ribosome biogenesis stress and ICD, the design of future combination regimens with immune checkpoint inhibitors should focus on delivery and sequencing. Ensuring sufficient intratumoral oxaliplatin exposure before or concurrent with the immune checkpoint blockade may be critical for creating a defined immunogenic window characterized by T-cell priming and maximal immune activation. To help localize oxaliplatin to the tumor, MB-FUS could play a critical role in this context by opening the BBB and increasing drug delivery into the TME, ensuring that oxaliplatin functions not only as a radiosensitizer but also as an immunomodulatory agent [71].

Moreover, platinum agents have been shown to influence the TME by modifying angiogenesis, extracellular matrix remodeling, and immune signaling pathways [20]. These changes may not only enhance radiosensitization but also counteract GBM-associated immune evasion. However, the immunomodulatory effects of platinum drugs remain insufficiently characterized in GBM, and further investigation is needed to determine how these effects can be harnessed to optimize combination therapies. A deeper understanding of how platinum compounds alter tumor oxygenation, vascular permeability, and immune cell activation may reveal novel mechanisms for overcoming the intrinsic resistance of GBM to conventional treatments.

Despite their substantial therapeutic potential, platinum-based drugs are subject to multiple resistance mechanisms that may significantly diminish their efficacy in GBM. Key mechanisms of resistance include reduced intracellular drug accumulation driven by altered uptake and efflux transporter activity, increased detoxification through upregulated glutathione and related enzymes, enhanced DNA repair pathways (notably *via* PARP and other repair proteins), and the suppression of apoptosis [72]. Additionally, non-coding RNAs such as microRNAs and long non-coding RNAs modulate drug sensitivity and resistance [73]. Importantly, these resistance mechanisms differ mechanistically from MGMT-mediated resistance to TMZ, suggesting that platinum agents may retain activity in TMZ-resistant tumors but remain vulnerable to their own distinct set of resistance pathways.

To overcome platinum resistance in GBM, several innovative strategies are being explored. One promising approach involves the development of platinum(IV) prodrugs, which exhibit improved cellular uptake and can be designed to co-deliver inhibitors of resistance pathways, such as histone deacetylase (HDAC) inhibitors or cathepsin B modulators, thereby suppressing DNA repair and enhancing apoptosis [74,75]. Modulation of non-coding RNAs, including knockdown of resistance-associated microRNAs (such as miR-106a), and regulatory proteins (such as interleukin-24) has also demonstrated the ability to resensitize GBM cells to platinum drugs by reducing drug efflux and restoring apoptotic signaling [73]. Combination strategies that simultaneously target multiple resistance mechanisms represent a rational and likely necessary direction for future development, with the potential to substantially enhance the therapeutic impact of platinum-based radiosensitizers in GBM.

Although numerous preclinical studies exist that focus on platinum-based radiosensitization in GBM, the translation of these preclinical findings to clinical practice remains elusive and requires rigorous validation through clinical trials. Table 2 summarizes key results from several clinically tested platinum nanoformulations in non-CNS cancers, but the dearth of such formulations that advance to clinical trials in GBM can be largely attributed to the challenge of crossing the BBB. Establishing the safety and efficacy of MB-FUS combined with platinum-based radiosensitizers in human subjects may play a large role in translating platinum radiosensitizers to the clinic. Clinical trials are also essential to determine the optimal parameters for FUS application, the appropriate drug dosages, and the potential side effects in patients. The success of these trials will be crucial in establishing MB-FUS as a viable treatment option for GBM.

Advanced delivery strategies can significantly reduce systemic toxicity and enhance targeting specificity, but to further improve the safety and efficacy of platinum radiosensitizers, administration methods, dose optimization, and treatment protocols need to be considered. Local delivery can achieve higher intratumoral drug concentrations with reduced systemic burden. However, if multiple drug treatments are required over a long period of time, it will likely be beneficial to optimize MB-FUS in combination with systemic administration of platinum therapeutics. Using fractionated or subtoxic dosing regimens can help maintain radiosensitization while minimizing cumulative toxicity, especially when coordinated with radiation timing to maximize synergistic antitumor effects. Personalized approaches, including dose adjustments based on renal function, tumor biology, or genetic markers as well as desensitization protocols for hypersensitivity can improve treatment safety and tolerability in patients [76].

Given the current RT + TMZ standard-of-care, understanding how platinum radiosensitizers integrate into this clinical regimen will also be essential. Platinum agents and TMZ generate distinct types of DNA lesions, engage different cellular repair pathways, and trigger non-overlapping resistance mechanisms. In MGMT-unmethylated GBM patients (who comprise ~70% of all GBM patients) where TMZ is less effective, platinum agents may likely retain significant activity due to their MGMT-independent mechanisms of action. Combining platinum compounds with RT and TMZ may therefore enhance overall DNA damage, overwhelm multiple DNA repair pathways, and potentiate radiosensitization more effectively than either drug alone. Thus, integrating platinum-based radiosensitizers into RT + TMZ regimens may be particularly beneficial for tumors with high DNA repair capacity, unmethylated MGMT, or treatment-resistant phenotypes. This combination warrants systematic evaluation in both preclinical and clinical settings.

## 8. Conclusion

The exploration of platinum-based radiosensitizers in GBM treatment offers a promising avenue to address the limitations of existing therapeutic modalities. By leveraging the radiosensitizing properties of platinum compounds such as cisplatin, carboplatin, and oxaliplatin, these therapeutics can enhance radiosensitivity in GBM through multiple mechanisms, including the induction of DNA damage, modulation of oxidative stress and apoptotic pathways, interaction with cellular signaling pathways, and the inhibition of DNA repair pathways. Notably, platinum-based radiosensitizers offer mechanistic and therapeutic benefits that distinguish them from other types of radiosensitizers such as kinase inhibitors, autophagy inhibitors, and ROS-amplifying agents. Unlike inhibitors that rely on pathway-specific mechanisms, platinum agents exert their effects through broad-spectrum multimodal mechanisms, and their high atomic number further enhances radiation-induced photoelectron production, a property unique to metal-based radiosensitizers. Platinum (IV)-based prodrugs also offer tunability, allowing co-delivery of radiosensitizers, targeting ligands, and immunomodulators within a single construct. Recent innovations, such as nanoformulations and cell-based delivery systems, further expand the potential of these compounds by improving tumor targeting and minimizing systemic toxicity, overcoming key challenges associated with GBM therapy.

Although these results are promising, more work needs to be done to adopt platinum drugs as radiosensitizers for GBM treatment clinically. Clinical validation of these preclinical findings along with further research into how platinum drugs could potentially synergize with immunotherapies and PARP inhibitors would greatly advance this field of research. Identification of predictive biomarkers for platinum radiosensitizers – much like how tumor HRD status informs PARP inhibitor sensitivity and GBM MGMT promoter methylation predicts TMZ response – will be crucial for advancing their clinical translation. Incorporating such biomarkers into trial design could enable more precise patient stratification, guide dose optimization, and accelerate the development of personalized platinum-based radiosensitizer regimens. In parallel, continued advancements in imaging and ultrasound technologies will be vital for enhancing treatment precision, monitoring therapeutic response, and minimizing collateral damage to healthy brain tissue [77]. Overall, the integration of nano-and cell-based formulations of platinum drugs into GBM treatment regimens represents a significant advancement in cancer therapy. By leveraging the unique properties of these delivery strategies for drug delivery, researchers are uncovering new possibilities to enhance the efficacy of radiation therapy. Future studies are expected to focus on optimizing these formulations and exploring their potential in combination with other therapeutic modalities, thereby paving the way for more effective treatment strategies in combating GBM.

## Figures and Tables

**Table 1:** Past and Ongoing Clinical Trials of Radiosensitizers for GBM Treatment.

Clinical Trial ID	Phase	Radiosensitizer	Radiosensitizer Mechanism	Study Population	Combination Therapy	Status/Outcome
NCT03463265	I/II	ABI-009 (nab-rapamycin/ albumin-bound rapamycin)	mTOR inhibition: impairs DNA damage repair and causes cell cycle arrest	Newly diagnosed GBM	ABI-009 + Radiation + TMZ	Completed; median overall survival of 13.3 months with no serious adverse events
NCT01849146	I	Adavosertib	WEE1 kinase inhibition: blocks the G2/M checkpoints, resulting in mitotic catastrophe	Newly diagnosed GBM	Adavosertib + Radiation + TMZ	Completed; unacceptable dose-limiting toxicity rate in combination with RT and TMZ
NCT01752491	I	Ascorbate	H2O2 generation: increases oxidative stress and DNA damage through double-strand breaks	GBM	Ascorbate + Radiation + TMZ	Completed; no dose-limiting toxicities observed, median overall survival of 18 months
NCT02378532	I	Chloroquine	Autophagy inhibition: increases susceptibility to RT-induced damage	GBM	Chloroquine + Radiation + TMZ	Completed; favorable tolerability
NCT00869401	I/II	Dasatinib	Src kinase inhibition: suppresses pro-tumorigenic effects, interfering with DNA repair, and inhibiting cell migration and invasion	Newly diagnosed GBM	Dasatinib + Radiation + TMZ	Completed; no improvement in patient outcomes
NCT00039494	II	Erlotinib	EGFR inhibition: reduces DNA damage repair and hinders tumor growth and proliferation	GBM	Erlotinib + Radiation + TMZ	Completed; no benefit in overall survival
NCT00402116	I/II	Enzastaurin (ENZ)	PKCβ and PI3K/AKT signaling inhibition: suppresses tumor cell survival and proliferation, and interferes with angiogenesis, enhancing RT-induced apoptosis	Newly diagnosed GBM	ENZ + Radiation + TMZ	Completed; combination is well-tolerated, median progression free survival of 10.6 months
NCT00509821	II	Enzastaurin (ENZ)	PKCβ and PI3K/AKT signaling inhibition: suppresses tumor cell survival and proliferation, and interferes with angiogenesis, enhancing RT-induced apoptosis	Newly diagnosed GBM with unmethylated MGMT promoter	ENZ + Radiation	Completed; 53.6% of participants with progression free survival at 6 months
NCT01591577	II	Lapatinib	EGFR and HER2 inhibition: impairs DNA damage repair and reduces radioresistance	Newly diagnosed GBM	Lapatinib + Radiation + TMZ	Completed; tolerable and safe regimen
NCT00305864	I/II	Motexafin gadolinium (MGd)	Redox modulation: disrupts tumor metabolism, increasing susceptibility to radiation	Newly diagnosed GBM	MGd + Radiation + TMZ	Completed; well-tolerated treatment, no improvement in median overall survival
NCT02871843	I	RRx-001	Promotes tumor oxygenation through nitric oxide release and vascular normalization: enhances effect of RT-induced DNA-damaging free radicals	Newly diagnosed GBM	RRx-001 + Radiation + TMZ	Completed; no dose-limiting toxicities observed
NCT04205357	I	Sulfasalazine	System ${\text{X}}_{\text{c}}^{-}$ inhibition: reduces cystine uptake in cancer cells leading to glutathione depletion	Recurrent GBM	Sulfasalazine + Stereotactic RT	Completed; results submitted but not available yet
NCT01465347	I/II	Trans Sodium Crocetinate (TSC)	Enhances oxygen diffusion to the tumor: overcomes hypoxia-induced radioresistance	Newly diagnosed GBM	TSC + Radiation + TMZ	Completed; no serious adverse effects, 12-month overall survival of 71.2%
NCT00302159	II	Valproic Acid (VPA)	HDAC inhibition: lowers ability to repair RT-induced DNA damage	GBM	VPA + Radiation + TMZ	Completed; median overall survival of 29.6 months, 86% of participants with overall survival at 12 months
NCT00731731	I/II	Vorinostat	HDAC inhibition: epigenetic modulation creates a more open chromatin structure, increasing DNA susceptibility to damage	Newly diagnosed GBM	Vorinostat + Radiation + TMZ	Completed; median time to tumor progression of 8.05 months, 54.6% of participants with overall survival at 15 months
NCT02432417	II	Chloroquine	Autophagy inhibition: increases susceptibility to RT-induced damage	GBM	Chloroquine + Radiation + TMZ	Withdrawn due to lack of funding
NCT04881032	I/II	AGuIX nanoparticles	Radiation dose enhancement: Gadolinium (high atomic number)-based Auger cascade.	Newly diagnosed GBM	AGuIX nanoparticles + Radiation + TMZ	Ongoing; study in progress
NCT03423628	I	AZD1390	ATM inhibition: disrupts DNA damage repair	Primary and recurrent GBM	AZD1390 + Radiation	Ongoing; study in progress
NCT03672721	I/II	Carboplatin	Forms crosslinks with DNA which prevents DNA replication and increases DNA damage	Relapsing GBM	Carboplatin + Radiation	Ongoing; study in progress
NCT03862430	II	NanO2^™^	Enhances tumor oxygenation: overcomes hypoxia-induced radioresistance	Newly diagnosed GBM	NanO2^™^ + Radiation + TMZ	Ongoing; study in progress
NCT04555577	I	Peposertib (M3814)	DNA-PK inhibition: blocks NHEJ-mediated DNA repair	Newly diagnosed MGMT unmethylated GBM	Peposertib + Radiation + TMZ	Ongoing; study in progress

**Table 2:** Clinically Evaluated Platinum-Based Nanoformulations.

Clinical Trial ID	Trial Phase	Nanoformulation	Platinum Drug	Delivery System	Study Population	Status/Outcome
NCT01861496	Phase I/II	LiPlaCis	Cisplatin	Liposome	Advanced or refractory solid tumors (Phase I), metastatic breast cancer, prostate cancer, and skin cancer (Phase II)	Completed; well-tolerated treatment, median overall survival of 50 weeks in metastatic breast cancer patients
NCT00004083	Phase II	SPI-77	Cisplatin	Stealth liposome	Recurrent ovarian cancer	Completed; stable disease in 4/5 patients, concerns regarding large lipid load and prolonged persistence of platinum in the body
NCT00102531	Phase I/II	SLIT Cisplatin	Cisplatin	Liposome	Osteosarcoma metastatic to the lung	Completed; well-tolerated treatment with minimal toxicities associated with intravenous cisplatin, 3/8 patients showed sustained benefit
NCT02817113	Phase I	NC-6004	Cisplatin	Micellar nanocomplex (PEG-poly(glutamic acid))	Head and neck cancer	Study terminated
NCT02240238	Phase I/II	NC-6004	Cisplatin	Micellar nanocomplex (PEG-poly(glutamic acid))	Advanced solid tumors, or non-small cell lung, biliary, and bladder cancer	Completed; well-tolerated treatment, median progression-free survival of 204 days observed with bladder cancer patients, tumor shrinkage and stable disease reported in 55% and 70% of solid tumor patients, respectively
NCT00355888	Phase I	MBP-426	Oxaliplatin	Liposome with transferrin receptor targeting	Advanced or metastatic solid tumors	Completed; favorable tolerability with thrombocytopenia as main dose-limiting toxicity

## Data Availability

No data was used for the research described in the article.
